# Supplementary material for: Mechanisms governing the pioneering and redistribution capabilities of the non-classical pioneer PU.1
Source: Nat Commun. 2020 Jan 21;11:402. doi: 10.1038/s41467-019-13960-2 (PMC6972792; doi:10.1038/s41467-019-13960-2)
Supplement: Supplementary file 7 — Source data [file 41467_2019_13960_MOESM7_ESM.zip › Source_Data/FigureS2/FigureS2B_MotifScanOutput/KmeansCluster8/homerResults/motif5.similar.html]

motif5

## Information for motif5

G
A
T
C
G
C
T
A
T
C
G
A
A
G
T
C
G
T
A
C
C
G
T
A
A
T
G
C
G
C
T
A
C
A
G
T
T
A
G
C
  
Reverse Opposite:  

A
T
C
G
G
T
C
A
C
G
A
T
A
T
C
G
A
C
G
T
A
C
T
G
T
C
A
G
A
G
C
T
C
G
A
T
C
T
A
G
  

|  |  |
| --- | --- |
| p-value: | 1e-85 |
| log p-value: | -1.975e+02 |
| Information Content per bp: | 1.654 |
| Number of Target Sequences with motif | 420.0 |
| Percentage of Target Sequences with motif | 27.78% |
| Number of Background Sequences with motif | 4327.0 |
| Percentage of Background Sequences with motif | 9.53% |
| Average Position of motif in Targets | 101.0 +/- 53.3bp |
| Average Position of motif in Background | 99.1 +/- 69.0bp |
| Strand Bias (log2 ratio + to - strand density) | -0.1 |
| Multiplicity (# of sites on avg that occur together) | 1.13 |
| Motif File: | file (matrix) reverse opposite |

### Similar de novo motifs found

|  |  |  |  |  |  |  |  |
| --- | --- | --- | --- | --- | --- | --- | --- |
| Rank | Match Score | Redundant Motif | P-value | log P-value | % of Targets | % of Background | Motif file |
| 1 | 0.923 | G C A T T C G A T C G A G A T C T G A C C T G A A T G C C G T A C T A G | 1e-77 | -177.488345 | 33.93% | 14.38% | motif file (matrix) |
| 2 | 0.922 | C G A T C T A G G A C T A C T G A C T G A C G T A C G T C G T A | 1e-72 | -167.952752 | 30.82% | 12.62% | motif file (matrix) |
| 3 | 0.937 | C G T A C G T A A G T C A G T C C G T A A G T C G C T A | 1e-66 | -153.381646 | 24.27% | 8.89% | motif file (matrix) |
| 4 | 0.900 | C A G T C A G T A T C G A C G T A C T G A C T G A C G T C A G T C G A T T A C G G C T A | 1e-59 | -137.190286 | 11.44% | 2.36% | motif file (matrix) |
| 5 | 0.881 | A G T C A C G T A C T G A C G T A C T G A C T G A C G T A C G T C G T A A T G C C G A T C A T G C T G A | 1e-55 | -127.181703 | 16.93% | 5.37% | motif file (matrix) |
| 6 | 0.830 | G C T A A C G T A T C G G A C T A C T G A T C G A G C T | 1e-45 | -105.368875 | 64.02% | 45.42% | motif file (matrix) |
| 7 | 0.885 | C A G T T G A C G C A T T A G C G C A T T G C A T G C A A G T C A G T C C G T A A T G C G T C A | 1e-42 | -97.901964 | 13.36% | 4.27% | motif file (matrix) |
| 8 | 0.834 | T C A G A G T C G C T A C T G A T C G A A G T C A G T C C G T A A G T C C G T A T C G A G C A T | 1e-31 | -72.319840 | 5.69% | 1.09% | motif file (matrix) |
| 9 | 0.795 | G A C T G C A T C T A G A T G C A G T C C T G A A G T C G T C A A C G T | 1e-20 | -47.032204 | 14.02% | 6.95% | motif file (matrix) |
